# An Ugly Enterocele, Operation and Cure

**Published:** 1887-08

**Authors:** Frank H. Tucker

**Affiliations:** San Augustine, Texas


### AN UGLY ENTEROCELE:—OPERATION AND CURE.

*By Frank H. Tucker, M. D., San Augustine, Texas.*

[For Daniel's Texas Medical Journal.]

The following case of Enterocele came under my care in May, 1883. The woman was twenty-eight years old, mother of one child. When seven months advanced with her second pregnancy, she jumped from a wagon in rapid motion, felt something give way, and after walking a few hundred yards a large mass presented at the vulva, and she became sick and faint, and thought she was about to abort. She sent for a mid-wife, who put her to bed, and the tumor disappeared. Upon assuming the upright posture the tumor appeared at the vulva again. When I was sent for, upon examination I found a large mass, that came down to the right of the uterus, posterior, and to the right of the bladder. I replaced the tumor, and told her I could do nothing for her, advised recumbent posture for next two months, and admonished her, and the midwife to be sure that the hernia was reduced when labor came on. She went to full term, and had a normal labor. Three months after her confinement she applied to me again, clamorous for some relief, as the tumor was very large, and could not be kept up. I tried Fowler's and many other pessaries but failed to prevent descent of intestine. I finally told her and her friends, that I had no guide for an operation, and explained fully the danger of an operative procedure, but would attempt to relieve her, with full consent of all concerned, after fully apprising her of the danger and probable failure.

In November of said year, I proceeded to operate in the following manner: Placed her on an inclined plane, head down, in Sim's position, had perineum retracted by a Sim's speculum, made an incision over tumor, carefully dividing tissues down to ring of hernia, the tumor was reduced, and the intestines held in place by armed rods of whale-bone in the hands of assistants. I then brought the ring together in a double fold, and stitched straight across the fold with a continuous suture, which left a considerable flap below the sutures to slough. I then removed a piece of the vagina a little larger perhaps than the protruded tumor over the hernia. I then passed a ligature around the circular opening, bringing it out near the point of entrance, drew the ends together, after the manner of an old fashioned work bag, until a very small circular opening was left, through which was inserted a drainage tube. I then packed all around the tube with absorbent cotton, previously dipped in boro-glycerine. Here ended the operation, —and one that was very difficult and unsatisfactory. I was not sure, that the intestines had escaped injury, and I know the peritoneum had been wounded in the operation.

Twenty-four hours after operation found patient suffering intense pain, temp 103°, vagina very sensitive, so much so, that I had great difficulty in removing the cotton packing, after removal however, and irrigation with warm carbolized water, the patient was more comfortable, a violent pelvic peritonitis followed, and her condition for ten days was very precarious; on the twelfth day opened a pelvic abscess that had pointed in Douglass' cul-de-sac on the right side, at the same time opened a labial abscess that had formed on left labium, caused perhaps from bruising by instruments, washed out abscess cavity daily with bi-chloride solution 1=4,000 strength. From this date patient steadily improved, and at the end of three months discharged perfectly well, and entirely relieved of the Entrocele.

While the final result in this case was good, I would be slow to do the operation again, yet I am inclined to believe the operation in more skillful hands would be justifiable.

